# Effects of Different Sugar Types on Longevity, Fecundity, and Nutrient Metabolism in *Sclerodermus guani* Xiao *et* Wu (Hymenoptera: Bethylidae)

**DOI:** 10.3390/insects17030315

**Published:** 2026-03-14

**Authors:** Zhen-Jie Hu, Shao-Qing Qiu, Bo Min, Xin-Jie Yao, Meng-Yao Jia

**Affiliations:** 1 College of Horticulture and Plant Protection, Henan University of Science and Technology, Luoyang 471000, China; 17516217231@139.com (S.-Q.Q.); 15036902210@163.com (B.M.); m19712586361@163.com (X.-J.Y.); m17629795100@163.com (M.-Y.J.); 2 Henan Key Laboratory of Insect Biology, College of Horticulture and Plant Protection, Henan University of Science and Technology, Luoyang 471023, China

**Keywords:** *Sclerodermus guani*, longevity, fecundity, nutrients, sugar diets

## Abstract

*Sclerodermus guani* Xiao *et* Wu, 1983 plays a significant role in the biological control of agricultural and forestry pests. To investigate whether different sugar types significantly affect the longevity, fecundity, and nutrient reserves of female *S. guani* adults, this study provided 1 mol/L solutions of sucrose, fructose, glucose, mannose, or trehalose under laboratory conditions, with a distilled water group serving as the control. The longevity and nutrient content of parasitoids were measured after varying feeding durations, while fecundity was assessed in preliminary experiments. The results demonstrated that prolonged sugar feeding significantly extended parasitoid longevity, with fructose, glucose, and sucrose exhibiting the most pronounced effects and no significant differences among them. Nutrient analysis revealed that sugar consumption significantly increased total carbohydrate content, slowed lipid depletion, and promoted protein accumulation. Sucrose, fructose, and glucose outperformed other sugars and the control in these aspects. Fecundity assays indicated that glucose and trehalose significantly shortened the pre-oviposition period and enhanced egg production. In conclusion, nutritional supplementation markedly improves the longevity and reproductive performance of *S. guani*, with 1 mol/L glucose identified as the optimal dietary source.

## Simple Summary

*Sclerodermus guani* Xiao *et* Wu plays a crucial role in the biological control of agricultural and forestry pests. However, its application is currently limited by low reproductive efficiency and short storage duration. In this study, we investigated the effects of different sugar diets on the longevity and fecundity of *S. guani*. Additionally, we measured the nutrient content in *S. guani* after feeding on various sugars for different durations. The results demonstrated that nutritional supplementation significantly enhanced both longevity and fecundity, with 1 mol/L glucose identified as the optimal sugar source.

## 1. Introduction

The parasitoid *S. guani* primarily targets wood-boring pests, including longhorn beetles such as *Monochamus alternatus* Hope, 1843, *Anoplophora glabripennis* (Motschulsky, 1853), and *Massicus raddei* (Blessig, 1872), and *Cossidae* Leach, 1815. These trunk borers cause substantial economic losses to global agriculture and forestry [1]. Conventional chemical control methods raise environmental concerns and risks of pesticide resistance, whereas biological control has gained research attention due to its eco-friendly nature. As an obligate parasitoid, *S. guani* demonstrates significant potential for biocontrol applications in forestry and agriculture. Artificial rearing is prerequisite for its field deployment, yet low reproductive efficiency remains a major constraint. Consequently, developing cost-effective mass-rearing techniques has become a key research focus in biocontrol applications [2]. Additionally, improving the short survival time of *S. guani* colonies during storage presents an urgent challenge for practical implementation.

The nutritional requirements of parasitoids are primarily derived from two sources: the uptake of nutrients from hosts during embryonic development, and the consumption of exogenous food sources during the adult stage to support longevity and fecundity [3,4,5,6,7]. *S. guani* can feed on hosts as well as non-hosts [8,9]. Many female parasitoid wasps exhibit host-feeding behavior by consuming host hemolymph, while also utilizing carbohydrate-rich liquids such as floral/nectar resources or honeydew [10,11,12]. The primary nutritional components of these dietary sources include fructose, glucose, mannose, sucrose, and trehalose, sugar concentrations in nectar range from about 10–70% *w*/*w* [4,6,13,14,15,16]. Trehalose—a disaccharide derived from insect hemolymph—represents a characteristic component of certain honeydew secretions [17]. These nutritional compounds significantly influence key biological parameters of parasitoids, including longevity, fecundity, and host-searching capacity [13,18,19,20,21,22,23,24,25]. Previous studies have demonstrated that whether a high-sugar diet can extend lifespan is closely related to the feeding patterns of the host. For instance, in parasitic wasps, post-feeding behaviors—such as active host searching and dispersal—may enhance fitness under certain conditions, while prolonged resting periods can reduce effective foraging time and ultimately shorten functional longevity [26,27]. However, the suitability of honeydew as a food source for parasitoid wasps has been shown to vary considerably [28]. While some studies report that honeydew can significantly enhance parasitoid longevity [20,29,30], other research has demonstrated that honeydew provides no benefit and may even reduce adult lifespan [31]. Sugar composition is one of the key factors determining the nutritional value of honeydew, and minor differences in parasitoid longevity can, to some extent, be attributed to variations in sugar profiles [23]. It is evident that different types of sugars exert varying effects on the growth, development, and reproduction of parasitoid wasps, which may negatively influence their efficacy in biological control [32]. In the dynamic balance between host and parasitoid populations, longevity is a key indicator of parasitoid fitness, playing a crucial role in the control of agricultural and forestry pest populations. The extension of the parasitoid’s lifespan may affect its offspring production [33]. So investigating the effects of different sugar diets on the development and reproduction of *S. guani* is crucial for enhancing its biocontrol efficacy and practical application potential.

Feeding on floral nectar and honeydew affects the nutrient content within parasitoid wasps [34,35,36,37]. The supplementation of these sugar-rich diets can be immediately utilized for metabolic purposes to generate energy. Related studies have shown that the consumption of sugars can alter the total sugar and fat levels in parasitoid wasps, slowing the rate of lipid depletion [36,37,38,39,40,41], and may even increase fat content [33]. For example, Visser et al. (2010) fed honey to 21 parasitoid species and found that lipid levels increased in five species, decreased in eleven species, and remained stable in five species [42]. In addition, stable isotope labeling experiments have shown that female *Nasonia vitripennis* can convert D-glucose, D-fructose, sucrose, and α,α-trehalose (the primary sugars consumed by adult parasitoids in nature) into palmitic acid, stearic acid, oleic acid, and linoleic acid [43].

This experiment will test how different types of sugars affect the lifespan, reproductive ability, and nutrient content of *S. guani*. The results will help address the two major issues of short storage time and low reproductive efficiency of *S. guani*, while also providing theoretical support for large-scale rearing techniques, field release strategies, and improving pest control effectiveness.

## 2. Materials and Methods

### 2.1. Insect Sources

The parasitoid wasps (*S. guani*) were obtained from Baiyun Industrial Co., Ltd., Jiyuan, Henan Province, China. All experiments were conducted using 48–72 h post-emergence mated females.

The host insects, second-instar larvae of the yellow mealworm (*Tenebrio molitor* Linnaeus, 1758), were purchased commercially (Luoyang Flower Market, China) and reared on a diet consisting of 80% wheat bran and 20% soybean flour, with carrots provided as a moisture source.

### 2.2. Preparation of Solutions

#### 2.2.1. Preparation of Sugar Solutions

Five types of sugars, including glucose (Tianjin Kemiou Chemical Reagent Co., Ltd., Tianjin, China), fructose (Tianjin Kemiou Chemical Reagent Co., Ltd., Tianjin, China), maltose (Sinopharm Chemical Reagent Co., Ltd., Shanghai, China), mannose (Sinopharm Chemical Reagent Co., Ltd., Shanghai, China), and trehalose (Sinopharm Chemical Reagent Co., Ltd., Shanghai, China) (all of the above are of analytical grade), were selected and each dissolved in distilled water to prepare solutions with a concentration of 1 mol/L. The 1 mol/L concentration was selected based on: (1) its ecological relevance to natural nectar/honeydew sugar concentrations (0.5–1.5 mol/L) [6]; (2) methodological consistency with previous parasitoid nutritional studies [24,34]; and (3) pilot experiments showing optimal feeding response at this concentration without adverse effects.

#### 2.2.2. Preparation of Sulfuric Acid-Anthrone Reagent

For the subsequent determination of sugar content in parasitoid wasps, the sulfuric acid-anthrone reagent was prepared. A total of 150 mL of distilled water was added to a 1 L flask, and 380 mL of concentrated sulfuric acid was carefully added while cooling. Approximately 750 mg of anthrone was dissolved in the diluted Sulfuric acid. The prepared reagent should be stored in a refrigerator at 4 °C [44].

#### 2.2.3. Preparation of Vanillin-Phosphoric Acid Reagent

For the subsequent determination of lipid content in parasitoid wasps, the vanillin-phosphoric acid reagent was prepared. A total of 600 mg of vanillin was dissolved in 100 mL of hot water, followed by the addition of 400 mL of 85% phosphoric acid. The mixture was thoroughly mixed and stored in the dark [45].

### 2.3. Determination of Sugar-Type Effects on Longevity

Fifty-four treatments (the subjects were fed five types of sugars and one pure water control, with nine different durations of exposure) were established by administering each sugar solution for durations of 0, 1, 3, 5, 7, 10, 15, 20, and 25 days. For each treatment, 30 unfed female wasps of uniform size were individually housed in glass tubes (75 mm length × 12 mm diameter), with cotton plugs sealing the openings. The tubes were maintained under controlled conditions (25 ± 1 °C, 60–80% relative humidity, and a 16L:8D photoperiod).

Each *S. guani* was provided with 100 μL of test solution daily via filter paper strips (10 mm × 50 mm), with fresh strips replaced every 48 h. The glass tubes were arranged in plastic trays lined with moistened towels. Mortality of the parasitoids was recorded daily until all wasps had died.

### 2.4. Analysis of Major Substance Contents in S. guani Under Different Sugar Treatments

Unfed, size-matched female wasps (48–72 h post-emergence) were selected and sampled for analysis at different time points (1, 3, 5, 7, 9, 10, 15, 20, and 25 days) after being subjected to five sugar treatments and two control treatments (water-only feeding and no feeding). For each treatment and time point, nine wasps were randomly selected for testing, with each substance content measured in triplicate. The methods followed the reference procedure [34].

#### 2.4.1. Protein Determination

Protein concentration was determined using a reagent kit from Shanghai Biological Engineering Co., Ltd. through the Bradford method (Shanghai Biological Engineering Co., Ltd., Shanghai, China), each treatment used a single female insect (the instrument used to test absorbance is: TU-1900, Beijing Puxi General Instrument Co., Ltd., Beijing, China).

#### 2.4.2. Determination of Total Sugars and Lipids

A single parasitic wasp was placed into a 1.5 mL centrifuge tube, and 50 µL of 2% sodium sulfate solution was added into the tube. The wasp was then crushed with a glass pestle, and the pestle was rinsed with 450 µL of chloroform-methanol (1:2). The wash solution (450 µL of chloroform-methanol) was collected into the centrifuge tube, and the sample was centrifuged. After centrifugation, 200 µL of the supernatant was transferred to a new test tube for total sugar analysis, and another 200 µL of the supernatant was transferred to a separate test tube for lipid analysis. All tubes were heated at 90 °C until all the solution in the lipid tube evaporated, leaving approximately 50 µL of solution in the sugar tube.

Lipid Determination: 40 µL of sulfuric acid was added to the tubes containing lipid precipitates, and the mixture was heated at 90 °C for 2 min. The tubes were then cooled on ice and mixed with 960 µL of vanillin-phosphoric acid reagent. After a 15-min reaction at room temperature, the absorbance was measured at 525 nm (the instrument used to test absorbance is: TU-1900, Beijing Puxi General Instrument Co., Ltd., Beijing, China).

Total Sugar Determination: Anthrone reagent was added to the sugar tubes up to the 1 mL mark, and the mixture was heated at 90 °C for 15 min. The tubes were then rapidly cooled on ice, and the absorbance was measured at 625 nm (the instrument used to test absorbance is: TU-1900, Beijing Puxi General Instrument Co., Ltd., Beijing, China).

### 2.5. Effects of Different Sugar Types on the Reproductive Capacity of S. guani

Newly emerged *S. guani* females (48–72 h post-emergence) were individually introduced into glass tubes (75 mm length × 12 mm diameter). Each tube was provisioned with filter paper soaked in one of five test sugar solutions. After 24 h of feeding, the parasitoids were allowed to parasitize pupae of *Tenebrio molitor* at a wasp-to-host ratio of 2:1. Oviposition quantity: The total number of eggs laid per female (oviposition quantity) was recorded over five consecutive days following the first observed oviposition event. Oviposition latency: The duration from complete host paralysis to the first observed egg deposition (oviposition latency) was measured.

### 2.6. Statistical Analysis

The effects of different sugar types and feeding durations on the longevity, pre-oviposition period, fecundity, and nutrient reserves (protein, total carbohydrate, and lipid content) of *S. guani* were analyzed using one-way ANOVA followed by Tukey’s HSD post hoc test for multiple comparisons. Two-way ANOVA was employed to assess the interactive effects between sugar types and feeding durations.

## 3. Results

### 3.1. Effects of Different Sugar Types on Longevity

**Figure 1:**
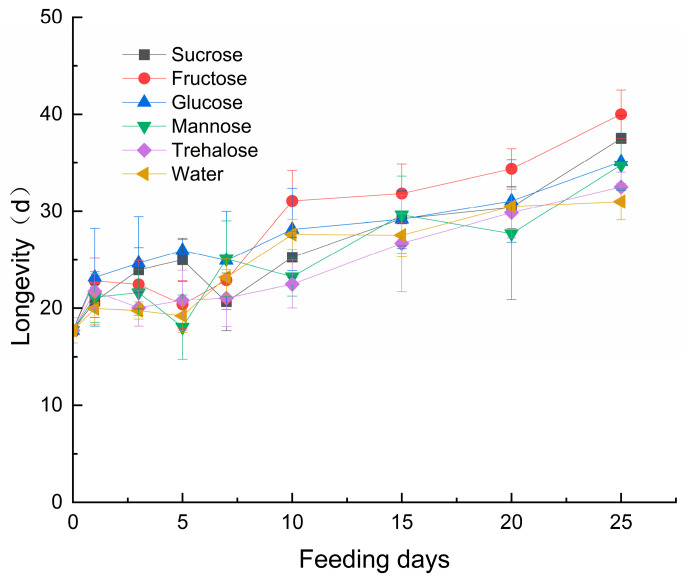
Effects of Sugar Types and Feeding Days on the Lifespan of *S. guani.* Note: Error bars represent the standard deviation; The y-axis intercept (0-day feeding duration) also represents the no-feeding control group data.

The longevity of *S. guani* was significantly influenced by sugar type (F_5,1566_ = 66.583, *p* < 0.001), feeding duration (F_8,1566_ = 656.146, *p* < 0.001), and their interaction (F_40,1566_ = 13.597, *p* < 0.001). During the 25-day observation period, parasitoid longevity exhibited an overall increasing trend with prolonged feeding duration (Figure 1). Fructose-fed groups consistently demonstrated significantly longer lifespans compared to other sugar treatments at 10, 15, 20, and 25 days (with the exception of glucose treatment). Parasitoids fed for ≥10 days showed significantly enhanced longevity compared to those with shorter feeding durations.

### 3.2. Effects of Different Sugar Types on Protein, Total Sugar, and Lipid Content in S. guani

**Figure 2:**
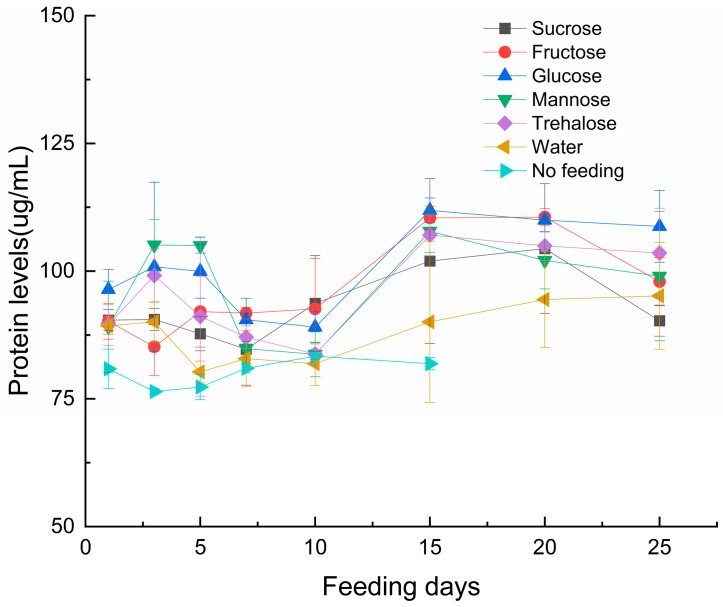
Effects of Sugar Types and Feeding Duration on Protein Content in *S. guani.* Note: Error bars represent the standard error.

Protein content (Figure 2): Significant differences were observed in the protein content of *S. guani* regarding sugar types (F_6,112_ = 235.592, *p* < 0.001), feeding duration (F_7,112_ = 167.624, *p* < 0.001), and their interaction (F_42,112_ = 29.822, *p* < 0.001). Glucose and fructose treatments resulted in higher protein content compared to other sugar types.

**Figure 3:**
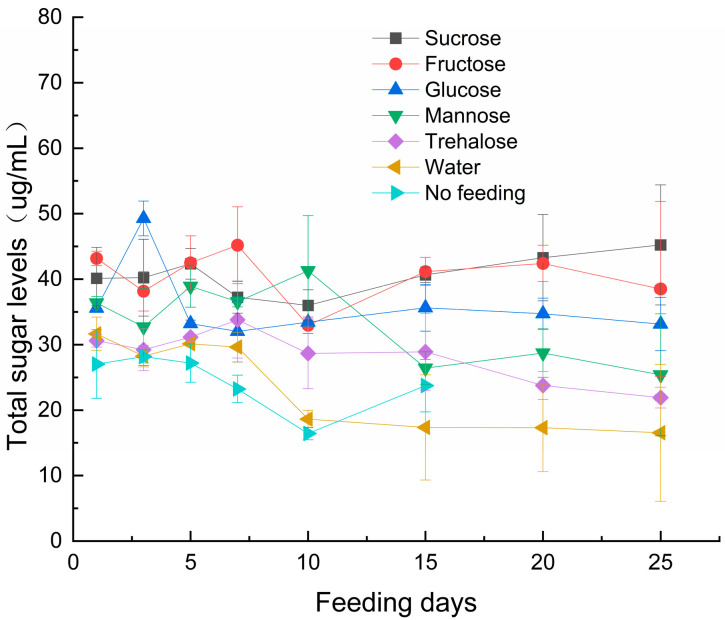
Effects of Sugar Types and Feeding Duration on Total sugar Content in *S. guani.* Note: Error bars represent the standard error.

Total sugar content (Figure 3) in the parasitoid was significantly influenced by sugar type (F_6,112_ = 55.328, *p* < 0.001), feeding duration (F_7,112_ = 7.215, *p* < 0.001), and their interaction (F_42,112_ = 2.708, *p* < 0.001. Fructose, sucrose, and glucose treatments led to a significant accumulation of total sugar content in the parasitoid wasps.

**Figure 4:**
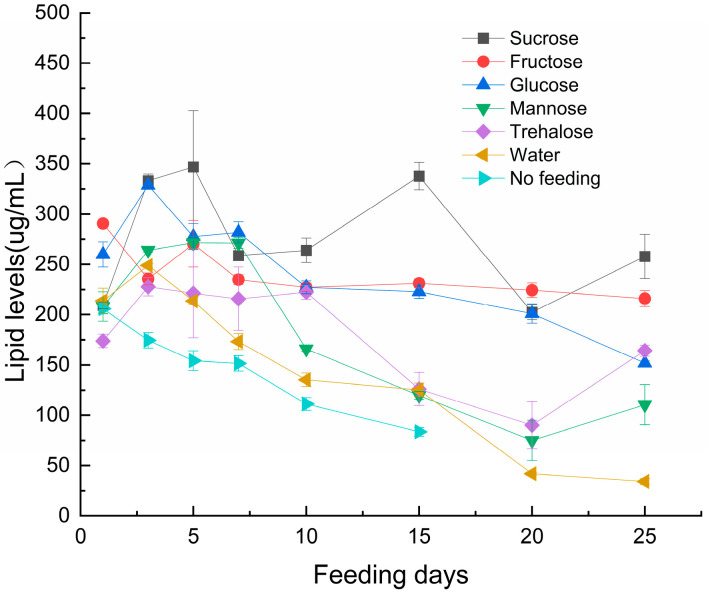
Effects of Sugar Types and Feeding Duration on Lipid Content in *S. guani*. Note: Error bars represent the standard error.

Lipid content (Figure 4) in the parasitoid was significantly affected by sugar type (F_6,112_ = 321.851, *p* < 0.001), feeding duration (F_7,112_ = 214.071, *p* < 0.001), and their interaction (F_42,112_ = 22.111, *p* < 0.001). Compared to the no-feeding control and water-only groups, sugar treatments (particularly fructose and glucose) significantly slowed the rate of lipid depletion (*p* < 0.05). However, no significant difference in lipid content was observed between the fructose and glucose groups (*p* > 0.05). The most rapid lipid depletion occurred in the no-feeding and water-only groups, followed by mannose and trehalose treatments.

### 3.3. Effects of Different Sugar Types on the Fecundity of S. guani

The sugar type significantly influenced both the oviposition quantity (F_5,174_ = 4.250, *p* < 0.001) and pre-oviposition period (F_5,174_ = 12.538, *p* < 0.001) of the parasitoid wasp. Glucose and trehalose treatments significantly shortened the pre-oviposition period compared to the water-only control, with glucose showing particularly pronounced effects.

**Figure 5:**
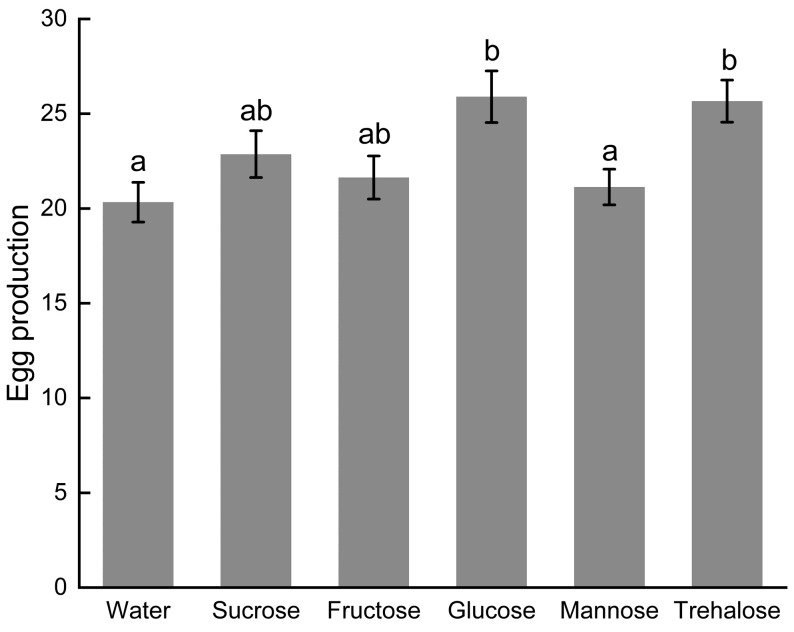
Effect of Sugar Types on the Oviposition of *S. guani*. Note: Error bars represent the standard error, different lowercase letters above the bars indicate significant differences at the 0.05 level.

**Figure 6:**
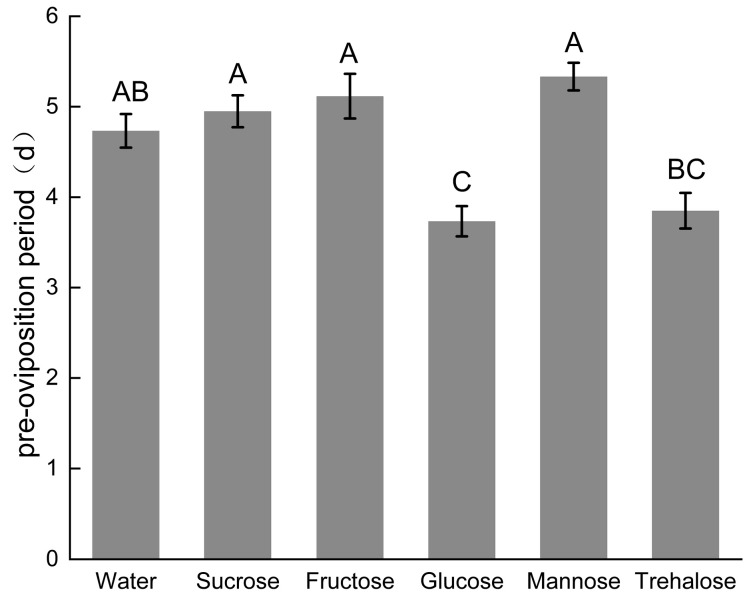
Effect of Sugar Types on the Pre-spawning period of *S. guani*. Note: Error bars represent the standard error, different uppercase letters above the bars indicate significant differences at the 0.01 level.

Our results demonstrate that different exogenous nutrients differentially affect the reproductive performance of *S. guani*. Glucose and trehalose significantly enhanced oviposition on *Tenebrio molitor*, with mean egg production reaching 25.90 ± 1.36 and 25.67 ± 1.11 eggs, respectively. These sugars also significantly reduced the pre-oviposition period to 3.73 ± 0.92 and 3.85 ± 1.08 days, respectively, indicating their potential to improve the reproductive capacity of *S. guani* (Figure 5 and Figure 6).

## 4. Discussion

This study systematically investigated the effects of different sugar nutrients on the longevity, fecundity and nutrient reserves (protein, total sugar, and lipid) of *S. guani*. The results demonstrated that sugar supplementation significantly extended female longevity, which aligns with the biological characteristics of synovigenic parasitoid wasps. During the 25-day observation period, the longevity of sugar-fed groups exhibited an increasing trend with prolonged feeding duration, supporting the continuous nutritional demand of synovigenic parasitoids.

Under long-term feeding (≥10 days), the fructose group consistently showed superior longevity compared to other sugars, though no significant difference was observed between fructose and glucose treatments. This may be attributed to their efficient metabolic pathways: fructose can directly enter cellular metabolism via an insulin-independent pathway [46], while glucose serves as a readily available energy source. In contrast, sucrose and trehalose require enzymatic hydrolysis into monosaccharides, and their conversion efficiency may vary due to individual metabolic differences. Additionally, mannose, which rarely occurs naturally in nectar or honeydew [13,47], may have different effects on parasitoid longevity. Longevity is a critical factor influencing the lifetime reproductive capacity of parasitoid wasps. Extending lifespan can translate into a higher total number of oviposition events, as females gain more time to locate hosts and lay eggs.

Regarding the results of fecundity measurements, the superior performance of glucose and trehalose in promoting egg production may be attributed to their rapid assimilation and efficient metabolic utilization in parasitoid wasps. Glucose, as a primary energy source, likely provides immediate ATP for oviposition-related activities. As for trehalose, the reason may be that it is the primary circulating sugar in the hemolymph of most insects [15]. It can be rapidly hydrolyzed into glucose (via trehalase), providing energy for oocyte development and vitellin synthesis [48].

Protein content exhibited non-linear changes with feeding duration, showing relatively low synthesis rates during the initial phase. In the 25-day trial, the glucose-fed group reached peak protein content at day 15, followed by the fructose treatment. The reason may be that the parasitic wasps were subjected to starvation treatment before feeding, which activated their metabolic compensation mechanism, thereby enhancing the protein-sparing effect of sugars [49].

Carbohydrates serve as essential energy substrates for insect physiological activities. Our results demonstrated significant accumulation of total sugars in *S. guani* following fructose, sucrose, and glucose feeding, indicating efficient metabolic utilization of these saccharides. The observed relationship between feeding duration and longevity indicates that carbohydrate reserves may influence longevity in this parasitoid wasp.

Sugar feeding did not result in significant net lipid accumulation. However, compared to the non-fed and water-only control groups, sugar treatments (particularly fructose and glucose) significantly attenuated the rate of lipid depletion (*p* < 0.05), suggesting that carbohydrate intake contributes to lipid reserve stabilization. Previous studies have reported that parasitoid wasps initiate fatty acid synthesis following sugar ingestion, with at least partial conversion of these metabolites [37].

Our 25-day observation period revealed no net increase in lipid content, likely reflecting a dynamic equilibrium between lipid synthesis and catabolic utilization. These findings indicate that parasitoid wasps employ efficient resource allocation strategies, though the underlying mechanisms require further investigation.

Sugar supplementation significantly enhanced oviposition capacity and reduced the pre-oviposition period in the parasitoid wasps, with particularly pronounced effects observed in the glucose and trehalose treatment groups. We found that these two sugar treatments also resulted in higher body protein content compared to other groups after just one day of feeding. However, the potential correlations among protein content, fecundity, and pre-oviposition period require further investigation. Furthermore, our analysis was limited to oviposition quantification and did not include comparisons of either the number or proportion of emerged adults, which warrants further investigation.

While our study primarily focuses on the dynamic changes in nutrient composition, the physiological effects of dietary sugars may extend to both immune function and gut microbiota. Carbohydrates may enhance immune capacity through mechanisms such as hemocyte proliferation [50], while sugar composition appears to regulate gut microbial communities, as evidenced by the promotion of beneficial symbionts by monosaccharides in social insects [51,52].Future investigations should integrate transcriptomic and microbiome analyses to elucidate these interactions, which are critical for optimizing the health of parasitoid insects in mass-rearing systems. Furthermore, we did not examine critical parameters including sugar concentration gradients, feeding frequency, or the precise conversion rates between lipids and carbohydrates across different sugar types; these parameters will all affect its results [11,43,53]. Future research incorporating biochemical analyses would be valuable to elucidate the dynamic processes of nutrient storage and utilization in these parasitoids.

## Data Availability

The original contributions presented in this study are included in the article. Further inquiries can be directed to the corresponding author.
